# Hepatitis B vaccination coverage in Germany: systematic review

**DOI:** 10.1186/s12879-021-06400-4

**Published:** 2021-08-14

**Authors:** Gyde Steffen, Ida Sperle, Thomas Harder, Navina Sarma, Sandra Beermann, Roma Thamm, Viviane Bremer, Ruth Zimmermann, Sandra Dudareva

**Affiliations:** 1 Department of Infectious Disease Epidemiology, Unit for HIV/AIDS, STI and Blood-borne Infections, Robert Koch Institute, Berlin, Germany; 2 Translational Epidemiology of DZIF, Department of Infectious Disease Epidemiology, Robert Koch Institute, Berlin, Germany; 3 Department of Infectious Disease Epidemiology, Unit for Gastrointestinal Infections, Zoonoses and Tropical Infections, Robert Koch Institute, Berlin, Germany; 4 Department of Infectious Disease Epidemiology, Immunisation Unit, Robert Koch Institute, Berlin, Germany; 5 Department of Infectious Disease Epidemiology, Unit for Crisis Management, Outbreak Investigations and Training Programmes, Focal Point for the Public Health Service, Robert Koch Institute, Berlin, Germany; 6 Centre for International Health Protection, Robert Koch Institute, Berlin, Germany; 7 Department of Epidemiology and Health Monitoring, Unit for Physical Health, Robert Koch Institute, Berlin, Germany

**Keywords:** Hepatitis B, Vaccination coverage, Germany, Populations at risk, Systematic review

## Abstract

**Background:**

Despite being considered as a low prevalence country for hepatitis B (HBV), some populations in Germany are at higher risk of infection. In the context of the World Health Organization’s (WHO) viral hepatitis elimination goals, a valid epidemiological data base is needed to plan and monitor the national response. Prevention strategies include general and targeted HBV vaccination programmes.

**Objective:**

The aim of this work was to estimate the HBV vaccination coverage (VC) in the general population (GP) and different population groups in Germany from available evidence and to identify current evidence gaps for future research.

**Methods:**

We conducted a systematic review on HBV VC in the general population and populations at high risk of HBV exposure or severe infection in Germany. We included eligible publications (01/01/2017 to 06/06/2020) from databases Embase, Pubmed and Livivo, from a previous scoping review (including data published 01/01/2005–17/03/2017), from the national surveillance system and screened the reference lists of all publications at full text level. Risk of bias was assessed using the Hoy et al. tool.

**Results:**

We included 68 publications of 67 studies and assigned them to one or more suitable population groups. Twenty-one studies contained data among children/adolescents and three among adults from the GP (VC 65.8–90.5% and 22.9–52.1%, respectively), one among travelers (VC 89.0%), 13 among immunocompromised populations (VC 7.8–89.0%), 16 among populations with occupational risk and 16 with non-occupational risk of HBV exposure (VC 63.6–96.5% and 4.4–84.5%, respectively).

**Conclusion:**

Comprehensive evidence at low risk of bias was identified for children/adolescents. However, 25 years after including HBV in the national immunisation schedule, VC in Germany is still below the 95%-goal defined by WHO. For people at occupational risk of HBV exposure, VC was mostly reported to be over the WHO goal of 80%, but quality of evidence was heterogenous and should be improved. For people at non-occupational risk of HBV exposure, evidence was sparse and of low quality. The low VC highlights the need for future research to plan vaccination programmes targeting these populations.

**Supplementary Information:**

The online version contains supplementary material available at 10.1186/s12879-021-06400-4.

## Background

Hepatitis B is a potentially life-threatening viral infection causing acute and chronic infection. In the World Health Organization (WHO) European Region, around 15 million people are infected with the hepatitis B virus (HBV). Annually, 56,000 persons die, mostly due to chronic HBV infection-related long-term sequelae like liver cirrhosis and hepatocellular carcinoma [1]. There is no specific treatment available for acute HBV infection, and treatment of chronic HBV infection can prevent the development of sequelae but mostly does not eradicate the virus. However, since the 1980s highly effective vaccines against HBV are available.

With a HBV prevalence of 0.3% found in the latest population-based survey *German Health Interview and Examination Survey for Adults* (DEGS1) Germany is considered to be a low prevalence country [1, 2]. Still, some populations in Germany may be at higher risk of HBV because of frequent contacts with infected blood or body fluids by occupational or non-occupational exposure or because they migrated from HBV endemic countries [3–6]. Higher prevalence among these groups has been reported [7].

Since 1995, the three, respectively four-dose (depending on the vaccine used) HBV vaccination in early infancy (0 to 14 months) and catch-up vaccination in non-vaccinated adolescents up to 18 years is recommended in the national immunisation schedule by the *German Standing Committee on Vaccination* (STIKO) [8] and are covered by German health insurance funds. Furthermore, HBV vaccination (including booster doses if applicable) is recommended for travelers after an individual risk assessment, for adults at risk of severe HBV due to immunodeficiency /immunosuppression or increased risk of occupational or non-occupational HBV exposure (e.g. intravenous drug use, changing sexual contacts) [9]. For adults in the general population not belonging to one of the above-mentioned indication groups, vaccination is not recommended. There is no legal requirement for HBV vaccination in any population group, including health care workers (HCW).

The European Vaccine Action Plan 2015–2020 defined HBV control through immunisation as one of its major strategic goals [10]. Furthermore, to reach the goal of eliminating viral hepatitis as a public health threat in Europe by 2030, the action plan for the health sector response to viral hepatitis in the WHO European Region, which was released in 2016, states worldwide vaccination goals to reduce transmission of HBV [11]. These include overall HBV childhood vaccination coverage (VC) of 95% with three doses of HBV vaccine, prevention of mother to child transmission and 80% VC in HCW. The prevention of HBV transmission associated with intravenous drug use is highlighted as an important element of the action plan, including control through vaccination. Meanwhile, the German government’s integrated strategy for human immunodeficiency viruses (HIV), HBV, Hepatitis C (HCV) and other sexually transmitted infections calls for data to improve the national response to the WHO elimination goals [12]. This strategy focuses on viral hepatitis control, and prevention strategies among groups at increased risk of acquiring and transmitting viral hepatitis. In this context, evidence-based knowledge about HBV VC in different population groups in Germany is essential to support targeted vaccination programmes in populations with vaccination gaps, and to monitor their implementation.

The aim of this work is therefore to summarize the VC in different population groups in Germany in light of the available evidence, and to identify evidence gaps as a target for further research.

## Methods

### Search strategy and review process

The systematic review followed a protocol registered in the Prospective Register for Systematic Reviews (PROSPERO; registration no. CRD42020186280). The search and reporting methods used were consistent with the guidelines in the Preferred Reporting Items for Systematic Reviews and Meta-analyses (PRISMA) statement [13].

The research question was “What is HBV immunisation coverage in Germany?”. According to the eligibility criteria we included publications of studies investigating the HBV VC in Germany with no restrictions regarding region or data collection setting and published in German or English language. We excluded studies with data collection ending before 01/01/2005.

We searched MEDLINE, Embase and Livivo on from 01 January 2017–19 May 2020 and updated this search on 06 July 2020. For details on the complete search strategy, see Additional file 1.

Abstracts and full texts were screened applying predefined eligibility criteria (see Additional file 2). When publications were identified that used the same data set, the publication with less information regarding VC was excluded.

We manually screened the reference lists of all publications included in full text-screening and the reports of the national surveillance data published by the Robert Koch Institute (RKI) at title/abstract- and full text-level. Moreover, we included the identified publications regarding HBV VC in Germany from a scoping review we previously conducted with a search time frame from 01 January 2005–09 March 2017 [14] and re-assessed them regarding eligibility.

Standardised forms were used to extract study characteristics and to assess risk of bias. We extracted the following data: study design, study population, region of data collection, number of study participants, setting, sampling frame and sampling of participants, study period, inclusion criteria, study instruments, demographics of the study population (age, sex), number of participants for outcome VC, definition of vaccinated participants and corresponding results for VC.

Screening and data extraction were done by two independent reviewers. Discrepancies were resolved by discussion.

For conference abstracts, we tried to identify the responding publication or contacted the authors for further information on study characteristics. Conference abstracts without any information on VC and study characteristics were excluded.

### Data analysis

Data was analysed by population group. We used the definitions of the study population according to the respective publication. Data was then allocated to one or more suitable pre-defined population groups including

- Children/adolescents from the general population (GP);
- Adults from the GP;
- People at risk of severe course of HBV infection due to immunosuppression: people living with HIV (PLWH), patients on haemodialysis or with other non-VH related underlying diseases;
- People at increased risk of occupational exposure: health-care workers (HCW), people working in facilities where HBV infected people are likely to be present;
- People at increased risk of non-occupational HBV exposure: household contacts of people living with viral hepatitis (PLWVH), people with a direct (born abroad) or indirect (born in Germany, one or both parents born abroad) migration background from HBV endemic countries, people at high risk of acquiring HBV through sexual contacts (e.g. men who have sex with men, MSM), people who inject drugs (PWID) and people in prisons and closed settings;
- Travelers.

The study instruments used to estimate the VC were categorised as i) serostatus (isolated positive anti-HBs), or medical record of serostatus; ii) vaccination card or medical record of vaccination; iii) medical record (of unspecified marker) and iiii) self-reported vaccination status. VC was calculated as the proportion of vaccinated participants among all participants (unless the denominator used in the respective publication was the number susceptible) and reported as percentages including 95% confidence intervals where available.

HBV VC was reported as coverage of complete or incomplete vaccination schedule according to the 2017 STIKO recommendations [15]. For children complete HBV vaccination schedule was defined as primary immunisation with at least three doses of monovalent vaccine or four doses of hexavalent vaccine. For adults, a complete HBV vaccination schedule was defined as primary immunisation with at least three doses of monovalent/bivalent vaccine. An incomplete vaccination schedule for children were defined as one to two doses of monovalent vaccine or one to three doses of hexavalent vaccine and for adults as one to two doses of monovalent/bivalent vaccine. The reported definition of complete/incomplete VC for each publication is stated in Additional file 3. A vaccination schedule was defined as “not specified”, when the number of vaccination doses received could not be assessed by the study instrument, when the vaccination doses received could not be assessed for the whole study population or when it was stated as “not specified” in the respective publication.

Current protection was defined based on STIKO recommendations for booster dose for respective indication groups (e.g. HCW) [8] and VC of current protection was reported separately.

When the VC was calculated (only) for the HBV susceptible population, this was indicated.

### Risk of bias assessment

For all studies, the Hoy et al. risk of bias tool for prevalence studies was used [16]. The tool incorporates ten items which assess internal and external validity of the studies, including representativeness of the data, sampling methods, mode of data collection, case definitions used, reliability and validity of the study instruments, and reliability of the calculations made. Each item was assigned a score of either zero or one. Studies with a total score of less than six points were categorised as being at high risk of bias.

## Results

### Selection of studies

**Fig. 1:**
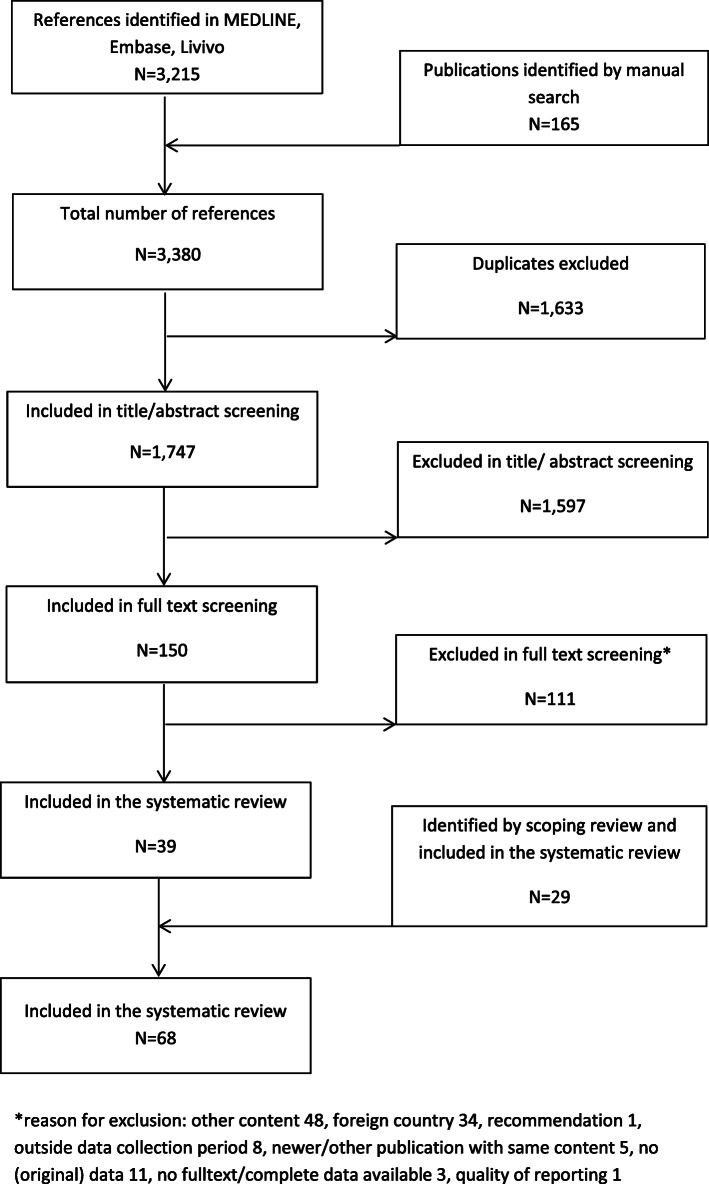
Study flow

We identified 3215 titles in electronic databases. One hundred sixty-five additional titles were found by manual search. After removal of duplicates, abstract and full-text screening, 41 publications were identified which contained relevant data on HBV VC in Germany. After removal of two non-informative conference abstracts, 39 publications were included in the systematic review [2, 17–54]. Additionally, 29 publications matching the inclusion criteria were added from the scoping review [55–83], giving a total of 68 publications in the final review. For details see Fig. 1.

### Study characteristics

Sixty-five of the included publications were articles in scientific journals, two were dissertations and one was a conference abstract.

The included publications reported data from 67 different studies. The nationwide population-based survey DEGS1 assessed VC using different study tools and the results were therefore published in two separate publications [2, 42].

All 67 included studies had a cross-sectional design, six of them additionally contained a cohort element [29, 36, 41, 48, 72, 77]. However, longitudinal data was not considered for this review.

In 27 studies, data was collected up to 2010. In three studies the data collection time frame was not reported, these studies were published in 2012 [59], 2018 [48] and 2019 [30].

Twenty-nine studies contained nationwide data, 16 contained regional data and 23 contained data from only one city. For three of the nationwide studies (reported in four publications), the sample was drawn from the German residential register [2, 40–42] and for two from other national registers [39, 49]. For one nationwide and one regional study, data from health insurance refund claims from the Association of Statutory Health Insurance Physicians (ASHIP) was analysed [17, 45]. For all other studies participants were recruited directly, or data came from existing data bases (e.g. from school/day care entry examinations, medical records, cohorts etc.).

In 28 studies, vaccination status was taken from vaccination cards, or from medical records in which this was documented. In two studies, health insurance data was analysed [17, 45]. In 13 studies, blood samples were tested for anti-HBs as an indicator of vaccination, or medical records were screened for serological results. In 20 studies, the vaccination status was self-reported. In two studies medical records were checked, but it was not clearly stated which parameters or indicators were used [55, 65] and for one study occupational physicians filled out an online questionnaire to report the VC among their patients [18]. For DEGS1, participants presented vaccination cards and anti-HBs was investigated in a sub-group [2, 42].

In 36 publications, coverage of the complete VC schedule (herafter referred to as complete VC) was reported. In 12 of them, coverage of incomplete vaccination schedule (hereafter called incomplete VC) was also stated. For the remaining 32 publications (31 studies including the 14 where VC was determined by serology), the reported VC was assessed as “not specified”. For 12 studies VC of current protection was calculated [34, 35, 52, 56, 59, 63, 78–83].

In one study, only VC among the susceptible study population was available [27]. In three further studies VC for the susceptible part study population was calculated separately (not presented here) [48, 50, 59].

The main study characteristics of the included publications are shown in Additional file 3.

### Hepatitis B vaccination coverage by population group

In five studies, HBV VC was investigated in different study populations, either as a single entity or as a sub-population (e.g. HIV-positive MSM) [29, 38, 50, 62, 64]. Where applicable, these studies were therefore allocated to more than one population group below [29, 38, 50].

**Fig. 2:**
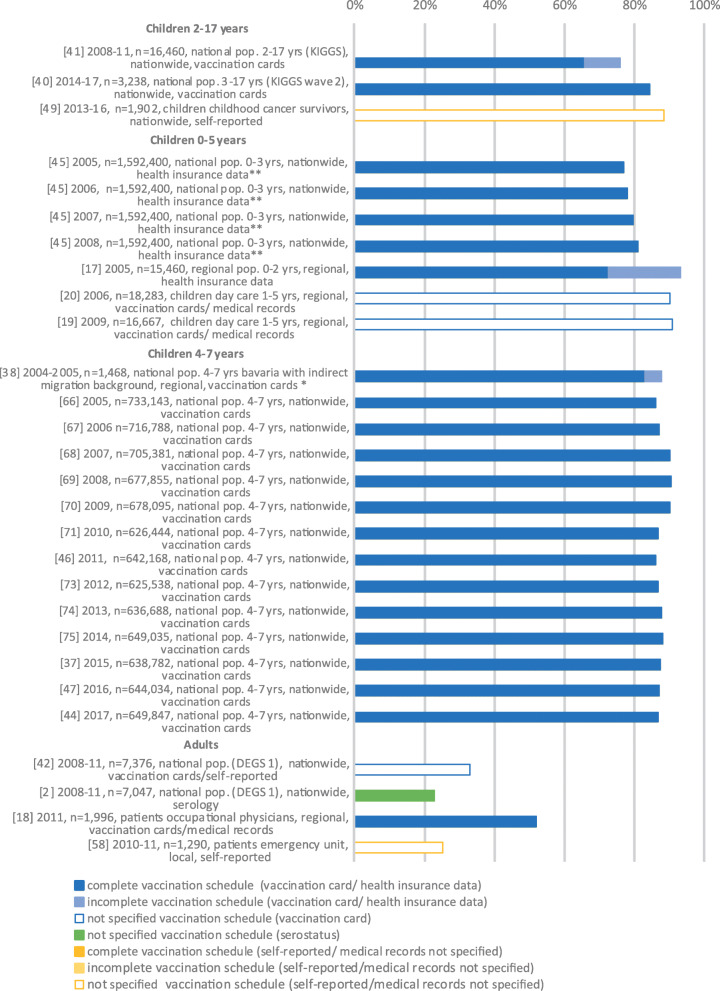
Vaccination coverage in Germany among the general population, %, 2005–2020. Reported are the reference number of the publication, year of data collection, sample size, study population, national/regional/local data collection and data collection tool. Vaccination coverage depicted as reported in the respective publication. *publication allocated to two population groups, **publication contains data stratified by year of data collection

Twenty-one studies reported data on HBV VC among children and adolescents (Fig. 2). Among these, 14 contained data from a mandatory national primary school entry medical and developmental check-up, showing a complete HBV VC between 83 and 90.5% (2005 and 2017) [37, 38, 44, 46, 47, 66–71, 73–75]. Two studies contained data from the national population-based health survey of children and adolescents (German Health Interview and Examination Survey for Children and Adolescents, KiGGS and KiGGS Wave 2), reporting a complete VC of 65.8% (2003–2006, 2–17 years) and 84.4% (2014–2017, 3–17 years) [40, 41], respectively. For KiGGS and KiGGS Wave 2 respectively, VC by age group was 74.8 and 85.9% (3–6 years), 68.1 and 89.3% (7–10 years), 59.6 and 85.4% (11–13 years), and 58.3 and 77.9% (14–17 years). For KiGGS, data for infants from 0 to 23 months was presented, showing a complete VC of 5.5% from 0 to 14 months and of 54.8% from 15 to 23 months.

Three studies estimated HBV VC in the adult general population. Using random samples of individuals aged 18 to 79 years, drawn between 2008 and 2011, the nationwide health survey DEGS1 reported an unspecified VC of 32.9% (through checks of vaccination cards, and self-reports) versus 22.9% (by serological marker) [2, 42]. A study of patients from an emergency room in Berlin performed in 2010/2011 showed an unspecified VC of 25.2% (self-reported) [58], and in another study complete VC of patients visiting occupational physicians was 52.1% [18].

**Fig. 3:**
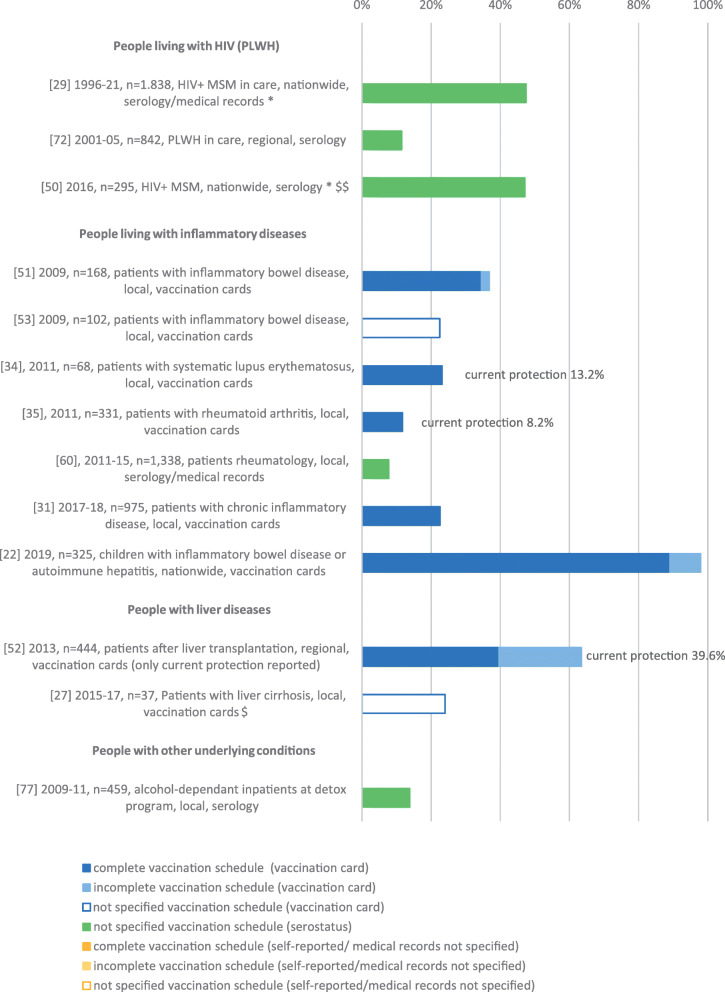
Vaccination coverage in Germany among populations at risk for severe HBV infection due to immunosuppression, %, 2005–2020. Reported are the reference number of the publication, year of data collection, sample size, study population, national/regional/local data collection and data collection tool. Vaccination coverage depicted as reported in the respective publication. *publication allocated to two population groups, $ HBV VC only in HBV susceptible people, $$ HBV VC also in HBV susceptible people available

Thirteen studies were related to immunocompromised populations (Fig. 3), including three in PLWH (two included only MSM) [29, 50, 72], seven in patients with autoimmune diseases [22, 31, 34, 35, 51, 53, 60], two in patients with liver cirrhosis resp. after liver transplantation [27, 52] and one among those undergoing alcohol-detoxification therapy [77]. The unspecified VC in PLWH was between 47.1 and 47.7% in HIV-positive MSM, and 11.5% in HIV-positive patients not restricted to MSM. In other immunosuppressed populations, the complete/not specified VC was between 7.8 and 89.0%, with a VC under 40% in nine of ten studies. The study reporting a VC of 89% was conducted in children and adolescents [22]. For three of 13 studies relating to immunosuppressed populations, the VC with current protection was reported [34, 35, 52].

**Fig. 4:**
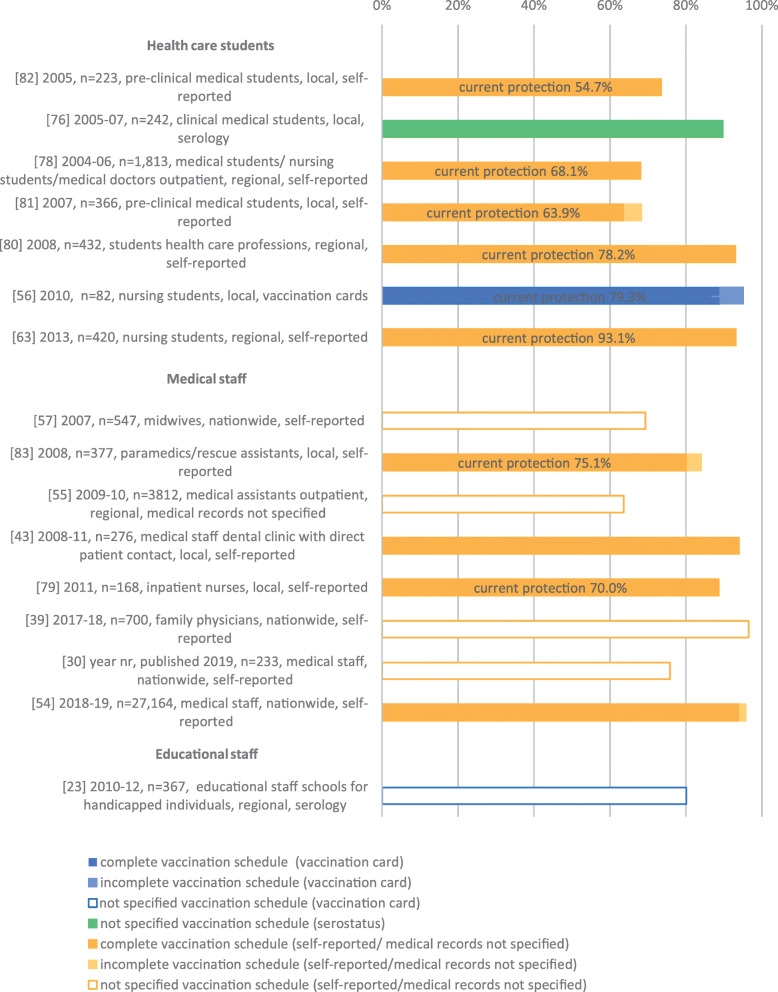
Vaccination coverage in Germany among populations at risk for occupational HBV exposure, %, 2005–2020. Reported are the reference number of the publication, year of data collection, sample size, study population, national/regional/local data collection and data collection tool. Vaccination coverage depicted as reported in the respective publication.

Sixteen studies investigated VC among populations at occupational risk of HBV exposure (Fig. 4). Seven studies looked at students studying to be a health professional (e.g. medical students, nursing students) and reported a complete/unspecified VC between 63.9 and 93.1% [56, 63, 76, 78, 80–82]. In eight studies the HBV VC was measured among hospital personnel including medical doctors, nurses, paramedics and other medical staff resulting in a complete/not specified VC between 63.6 and 96.5% [30, 39, 43, 54, 55, 57, 79, 83]. One study estimated the not specified VC among educational personnel in schools for handicapped individuals at 80.1% [23]. For eight studies, VC with current protection was reported [56, 63, 78–83]. In one of them, a study among health care students, current protection was higher than 80% (93.1%) [63].

**Fig. 5:**
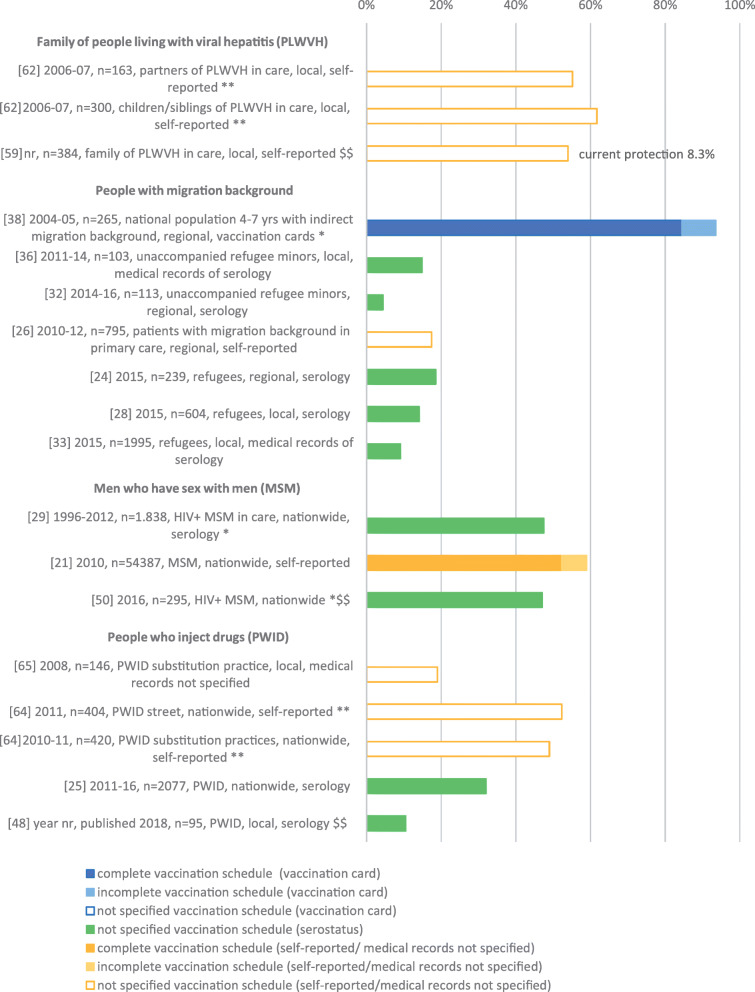
Vaccination coverage in Germany among populations at risk for non-occupational HBV exposure, %, 2005–2020. Reported are the reference number of the publication, year of data collection, sample size, study population, national/regional/local data collection and data collection tool. Vaccination coverage depicted as reported in the respective publication. *publication allocated to two population groups, **publication contains data on subgroups of the same population group, $$ HBV VC also in HBV susceptible people available

Seventeen studies considered people at increased risk of non-occupational HBV exposure (Fig. 5). Two studies were conducted among household contacts of PLWVH; these reporte a not specified VC of 54.0 and 55.2% in family and partners [59, 62], and 61.7% in children and siblings of PLWVH [62]. For one study, the VC with current protection was reported (8.3% of family members with known anti-HBs titre > 10 IU/L) [59].

Seven studies reported outcomes among people with a migration background (either directly or indirectly) from HBV endemic countries. One of these studies was a subanalysis of data from the school entry examination in Bavaria [38]. Two studies measured a not specified VC of 4.4% resp. 14.9% in unaccompanied refugee minors (UAM) [32, 36], and three a not specified VC between 9.1 and 18.6% in adult refugees [24, 28, 33]. For patients in a general medicine practice with a direct or indirect migrational background, the not specified VC was 17.4% [26]. The complete VC in pre-school children with an indirect migrational background (born in Germany, one or both parents born abroad) was 84.5% [38].

Three studies were conducted among MSM. One of them reported results from an internet-based, survey showing a complete VC of 52.3% [21]. The two other studies were conducted among HIV-positive MSM (see above) with a VC between 47.1 and 47.7% [29, 50].

In four studies data was collected among PWID. The not specified VC was between 10.5 and 52.5% in drug consumption facilities and in consumption places on the street [25, 48, 64], and between 19.0 and 49.0% in opioid substitution treatment centres [64, 65].

Regarding travel-related indication for HBV vaccination, one study was identified reporting an unspecified VC of 89.0% in a web-based survey among travelers to Hepatitis A (HAV) endemic countries [61].

For other populations at non-occupational risk for HBV exposure (e.g. prisoners, sex workers), no studies were identified.

The VC by population group including main study characteristics is illustrated in Figs. 2, 3, 4 and 5.

### Risk of Bias

Risk of bias was assessed as low in 51 of 68 publications (score 6 20%, score 7 23%, score 8 16%, score 9 16%, score 10 25%). In the remaining 17 publications risk of bias was assessed to be high (score 3 24%, score 4 29%, score 5 47%). All publications among children/adolescents and adults in the GP were at low risk of bias. Six of 16 publications on populations at occupational risk for HBV were at high risk of bias, whereas the proportion was 8/16 for publications on populations at non-occupational risk for HBV. Important weaknesses of publications at high risk of bias were the use of self-reporting (*n* = 7), serology (*n* = 8) or medical records with unclear parameters (*n* = 2) as study instruments, lack of a proper case definition for complete/incomplete HBV immunisation (*n* = 12), as well as weak methods used to represent the target population (insufficient national representation *n* = 12, inadequate sampling frame *n* = 13, inadequate sampling *n* = 8). For details see Additional file 4.

## Discussion

This systematic review gives a comprehensive overview of the currently available evidence on HBV VC in different population groups in Germany.

High coverage of universal childhood immunisation is the most important means by which to control HBV, by eliminating the risk of transmission before risk behaviour even starts. Consequently, the target VC for this group as determined by the WHO is 95%.

In this review, we identified a comprehensive evidence base for children and adolescents which was overall at low risk of bias. Nevertheless, the results of KiGGS (2003–2006) showed that despite a recommendation being in place since 1995, the coverage of timely protective HBV vaccination in infants from 15 to 23 months was low (54.8%) [41]. KiGGS Wave 2 conducted between 2013 and 2018 reported a considerably higher HBV VC in all age groups between 3 and 17 [40]. Data on timely protective HBV vaccination schedules are currently being analysed. The gap in VC between the two surveys shows the slow increase in VC after the introduction of HBV vaccination in the national immunisation schedule. Still, the VC in KiGGS Wave 2 remained below the WHO 95%-VC goal in all age groups; and VC decreased with age, to under 80% among adolescents between 14 and 17 years. The poorer VC among adolescents is most likely associated with their year of birth (i.e. born before inclusion of HBV in the immunisation schedule), but also shows insufficient implementation of catch-up vaccination programmes for this age group, who may then be put at risk when entering into sexual relationships in adolescence.

The data from KiGGS Wave 2 correlates with data from the yearly nationwide examination of children entering primary school between 2014 and 2017, where the mean VC in four to seven-year olds was within the same range. However, in this yearly survey the VC never reached the 95%-goal and, more importantly, the VC did not significantly increase over the years [37, 38, 44, 46, 47, 66–71, 73–75]. In the school entry examinations, the mean VC for the simultaneously scheduled diphtheria vaccination was higher compared to HBV in all years (95.6% for diphtheria versus 88.0% for HBV). This illustrates the missed opportunity of timely HBV vaccination and delay of this specific vaccination to older age.

For the adult general population, the evidence was at low risk of bias. HBV catch-up vaccination in adults is currently only recommended for selected populations, which results in a low VC among the adult population overall. Limitations of these findings include that data from the population-based survey DEGS1 was outdated and may not represent the current situation.

There was only one publication regarding HBV VC in travelers to HAV endemic countries. VC was high, but the results might be biased, as the survey was online and the demographics of the study population were not reported.

Evidence for people at risk of severe HBV due to immunosuppression, except PLWH, was extensive and overall at low risk of bias. Nevertheless, most identified literature relates to clinical populations with autoimmune diseases. VC in adults was low to moderate among this group despite an existing need for access to health care and a HBV vaccination recommendation. Reasons for low coverage in this group, including alarmingly low current protection, remain unclear. The high VC in children and adolescents with inflammatory bowel disease or autoimmune hepatitis might be a result of universal vaccination recommendations, rather than one relating specifically to HBV [22]. Moreover, in this study, a positive attitude among parents towards vaccination was reported.

HBV vaccination for populations at increased occupational and non-occupational risk of HBV exposure is the second pillar of HBV prevention in Germany [9]. In contrast to populations at occupational risk, for other populations indicated for HBV vaccination, no VC goal has been defined by WHO. Nevertheless, vaccination is recommended by German STIKO. Large VC gaps in these groups may hinder the success of HBV elimination in Germany and should, therefore, be recognized at an early stage and considered in prevention strategies.

Comprehensive evidence of mixed quality was identified for people at occupational risk for HBV exposure. The large number of identified publications regarding HCW reported a wide range of VC, but it must be noted that the study populations were heterogeneous and evidence varied in terms of risk of bias. VC was often self-reported, resulting in a lack of reliability [84, 85] and a potential overestimation of the real VC. However, we assume that HCW are more aware of HBV and HBV-Vaccination compared to general public, therefore are not as prone to recall bias as has been reported in other studies. Still, most studies were conducted at local or regional level, thus only reflecting local prevention efforts of single hospitals, and the representativeness of the results may be limited. Since 2016, the RKI has been conducting a yearly nationwide online-survey on Influenza vaccination uptake among HCW in German hospitals. In 2019, data on HBV vaccination was also collected [54]. This survey and also three quarters of the other studies, reported a VC fulfilling the WHO goal of 80%. Moreover, for the majority of studies with low VC, data collection started before 2010, matching the increase of VC over time for the GP. In half of the conducted studies, in addition to the not specified/complete VC, the VC of current protection was also surveyed, which was below 80% for seven of eight studies. Overall, primary immunisation seems to be adequate in HCW but current protection is insufficient. Patterns in VC among different occupational groups could be identified.

Large evidence and VC gaps were identified among populations at non-occupational risk for HBV exposure. The number of conducted studies was particularly low for household contacts of PLWVH, people who migrated from HBV endemic countries, MSM and PWID. No studies were available on prisoners or sex workers.

The available evidence and the VC of household members of PLWVH was low (not specified VC 54–61%), but VC increased when measured in susceptible household members only (73–84%) [59]. However, current protection was reported to be below 60%. However, VC was self-reported and only 8% of persons currently protected knew their antibody titre.

Regarding people with a direct or indirect migration background from HBV endemic countries, evidence was divided by data for newly arrived refugees and for persons already living or born in Germany. The available evidence for refugees was at high to intermediate risk of bias and included local/regional studies conducted in reception centres. Overall HBV VC among refugees was below 20% with a minimum of 4.4% [24, 28, 32, 33, 36]. HBV VC was measured in study populations with different compositions in terms of country of origin, mostly from Sub-Saharan Africa, Syria and Afghanistan, which may explain the variation in VC [86]. The VC among children born in Germany but with either one or both parents with a migration background did not differ from the VC among children with parents born in Germany, when looking at data from pre-school examinations [27]. These children might be sufficiently covered by the universal vaccination recommendation for children. However, insufficient language mediation was a limitation of this study, so children of migrants with poor German language skills may have been underrepresented. A survey conducted in people with a migration background seeking care in a general practice reported a VC of only 57.1% in under 20-year olds decreasing with age, demonstrating a vaccination gap especially in the age group who should have been covered by the universal childhood immunisation program [26]. Limiting the validity of the data, this study was at high risk of bias and the study population was a convenience sample of individuals with any migration background, country of birth and/or length of stay in Germany. Therefore, conclusions regarding the population of persons with a migration background from HBV endemic countries in Germany, as well as persons with a specific migration background, cannot be drawn based on the evidence included in this review.

For MSM the evidence was limited and of mixed quality, and the reported VC was low (between 47 and 52%) [21, 29, 50]. The VC did not increase over time and, surprisingly, was not higher in the two studies among HIV-positive MSM. A limitation was the use of self-reported vaccination status in two of the three studies. In the Europe-wide EMIS-2010 survey among MSM, the complete VC was higher in Germany (52%) as compared to the European average (44.7%) [21]. In this study, vaccination uptake was higher in MSM who were affected by universal vaccination recommendations, as well as in MSM for whom specific vaccination recommendations existed. This matched the result that being “out” was correlated with being vaccinated in older MSM, but not in younger MSM, who were covered by universal childhood immunisation.

For PWID, the limited evidence on HBV VC was at high risk of bias and the VC varied substantially between studies. In two smaller local studies, the not specified VC was noticeably lower (10.5 and 19%, respectively) [48, 65] than in the two larger, nationwide studies (32–52%) [25, 64]. The wide range of VC among PWID might be due to specific characteristics of the study populations, including age, with younger people having been covered by infant vaccination which has been in place since 1995, whereas older PWID could only benefit from risk group vaccination. Furthermore, differences at local level such as specific campaigns may explain the variation, which has also been described by Haussig et al. [25]. Moreover, in this study, self-reported HBV vaccination status and serological results were compared by participant and only matched in 45% of cases. Taking this into account, VC might also be overestimated in the study of Mone et al. [64]. Low HBV VC among PWID was observed not only in street-based and low-threshold drug services, but also in opioid substitution therapy clinics (OST) [25, 65].

However, as in all populations with an increased risk for HBV exposure, a considerable proportion of the study population may have already been infected with HBV (i.e. no longer susceptible) and, therefore, have no indication for HBV immunisation. Unfortunately, this was only addressed by four studies in different populations, so that a systematic comparison was not possible.

The cut-off chosen to classify studies as high vs low risk of bias is not part of the original risk of bias tool by Hoy et al. However, the idea here was to provide the reader with a rough estimate on risk of bias in the identified studies. In this review, data on populations for whom vaccination is recommended often came from smaller, local studies with convenience samples. This may have biased the results and also limited the representativeness. Moreover, statistically reliable evidence on HBV VC among subgroups (e.g. groups of people with a certain migration background) cannot be derived from these samples. Many of the studies obtained data on vaccination status from self-reports, which have low validity and lead to an overestimation of VC [85, 87–89]. Also, results obtained from serology without further information on past immunisation only provide information on a current titre. They do not provide any information about long-term protection received by complete primary immunisation and booster doses. Thus, even fully vaccinated people may not have any detectable titre at the moment of investigation [90, 91]. Moreover, in some publications the definition of complete or incomplete vaccination status was not reported (and not all differentiated between incomplete and complete vaccination), which may have resulted in an overestimation of VC and must be considered when interpreting the results. The assumption that the exclusion of children without vaccination cards from the VC analysis derived from school entry examinations might result in an overestimation of VC was refuted by Rieck et al., who validated the results by comparison with health insurance billing data [92]. In terms of comparability of studies, the study populations and settings varied within the defined population groups and, as described above, data collection tools and definition of complete vaccination differed between the studies.

In conclusion, the population-based surveys in the GP as well as the pre-school examinations provide continuous, extensive data and can serve as a monitoring tool for the national vaccination programme. The DEGS1 update gern-study (*Gesundheits- und Ernährungsstudie in Deutschland)* is already planned for 2022 and will update the evidence on HBV VC among the adult general population. People with a direct or indirect migration background from HBV endemic countries will be recruited representatively and, due to a newly developed tool [93], more sufficiently than in DEGS1. However, other populations at high non-occupational risk for HBV exposure are still underrepresented in the population-based surveys, and targeted studies among these groups are lacking. In an effort to address this, in 2021 the RKI initiated a pilot study DRUCK 2.0 to monitor HIV, HBV, Hepatitis C (HCV) and Syphilis among PWID, and will generate nationwide data on HBV VC in PWID from blood samples every 2 years from 2022 onwards. A pilot for a nationwide cross-sectional study on HIV, HBV, HCV and other infectious diseases among homeless populations has also recently been conducted and will be rolled-out in 2022/2023. Still, there was no evidence identified on VC in people in prisons or sex workers. When planning studies in these populations, a participatory design should be chosen including involvement of communities and sufficient culture- and language mediation. Recruitment designs like respondent driven sampling can increase the representativeness of small, local studies. In contrast to that, the surveillance of VC in German HCW provides a large data pool. However, data could possibly be supplemented by a more robust data collection tool.

Given the identified VC in the included studies, more work is needed to improve the overall HBV VC in Germany, in order to reduce transmission and halt the HBV epidemic. One reason for missed or delayed HBV vaccination among children is mistrust among parents in HBV vaccination in general as well as in the hexavalent vaccine. This vaccine is recommended in early infancy in Germany for protection against diphtheria, tetanus, pertussis, hepatitis B, polio, and *Haemophilus influenzae* type b. While over 95% of parents in a survey [94] agreed to the vaccination of their children against poliomyelitis and tetanus, this proportion decreased to 81% for HBV. Thirty-three percent of parents declined the 6-fold vaccination, which led to postponed or missed HBV vaccination opportunity. Reasons given by parents for not vaccinating their children included the perception, that HBV was not dangerous for their child, fear of side effects in early infancy and long-term vaccine associated damages. Therefore, paediatricians should provide information to parents about HBV and HBV vaccination, advocate against postponing vaccinations and actively use catch-up vaccination opportunities for children that were not vaccinated in the first 14 months of age. As most of the GP in Germany seek health care at general practitioners and are also usually vaccinated there, these doctors should be encouraged to promote HBV vaccination, and reported barriers such as vaccine shortages and cost-coverage problems should be reduced [39]. Counselling should always include an assessment of the personal risk. This might also increase general awareness regarding HBV transmission risks, and sensitize persons at high risk for either HBV exposure or a severe course of infection. In Germany, equal access to health care facilities (with full coverage of costs even for persons without health insurance) and non-stigmatizing counselling are the basis for reaching the whole population. Educational material in different languages is essential in order to ensure accessibility for persons without sufficient German language skills. For PWID, on-site options for vaccination in low-threshold settings and/or as part of harm reduction services, including the “don’t ask, vaccinate”-strategy, and accelerated immunisation schedules, should be offered [95]. Incentives could help to encourage PWID to get vaccinated [96]. The future monitoring study DRUCK 2.0 can help to target vaccination programs and monitor vaccination uptake among PWID.

As vaccination is the most successful prevention measure implemented in the response to HBV, the gaps in evidence on HBV VC in Germany should be filled, and HBV vaccination progress should be continuously evaluated. The reasons for low VC among different population groups should be fully understood in order to manage targeted vaccination campaigns, tackle vaccination gaps and drive HBV elimination in Germany.

## Supplementary Information

**Additional file 1.** Inclusion criteria. Criteria for inclusion in the systematic review.

**Additional file 2.** Search strings. Search strings for search in electronical databases.

**Additional file 3.** Table of study characteristics. Study characteristics of included publications.

**Additional file 4.** Table of risk of bias assessment. Risk assessment of included publications.

## Data Availability

The datasets supporting the conclusions of this article are included within the article and its additional files.

## Abbreviations

DEGS1: German Health Interview and Examination Survey for Adults
GP: General population
HAV: Hepatitis A virus
HBV: Hepatitis B virus
HIV: Human immunodeficiency virus
KiGGS: Study on the health of children, adolescents and young adults in Germany
MSM: Men who have sex with men
PLWH: People living with HIV
PLWVH: People living with viral hepatitis
PWID: People who inject drugs
STIKO: German Standing Committee on Vaccination
VC: Vaccination coverage
VH: Viral hepatitis
WHO: World Health Organization
HCW: Healthcareworkers
