# Supplementary material for: Hepatitis B vaccination coverage in Germany: systematic review
Source: BMC Infect Dis. 2021 Aug 14;21:817. doi: 10.1186/s12879-021-06400-4 (PMC8364709; doi:10.1186/s12879-021-06400-4)
Supplement: Supplementary file 1 — Additional file 1. Inclusion criteria. Criteria for inclusion in the systematic review. [file 12879_2021_6400_MOESM1_ESM.pdf]

Study selection criteria

Inclusion criteria	Exclusion criteria
Time frame data collection:<br>data collection finished after 1/1/2005<br><br>Geographic location:<br>data collection in Germany<br><br>Setting:<br>no restrictions<br><br>Participants:<br>no restrictions<br><br>Reported outcomes:<br>HBV vaccination coverage in humans	Time frame data collection:<br>data collection finished before 1/1/2005<br><br>Geographic location:<br>no data from Germany<br><br>Reported outcomes:<br>no report of vaccination coverage
Time frame publication:<br>published after 01/01/2005<br><br>Study design:<br>• original study data of observational studies<br>o cross-sectional studies<br>o cohort studies<br>o cases of case-control studies<br>o case series<br>• surveillance data<br>• secondary/registry data<br>• meta-data from systematic reviews<br><br>Type of publication:<br>Original work with clear origin of analysed data<br>• articles in scientific journals<br>• reports<br>• dissertations<br>• conference abstracts including data on research question plus additional information from author<br><br>Peer review:<br>no restriction	Time frame publication:<br>Published before 01/01/2005<br><br>Study design:<br>Expert opinions, narrative reviews, editorials, comments<br><br>Type of publication:<br>• Non-published data<br>• Abstracts: No full text existing or available for study team, no relevant data on outcome in the abstract, no consultation of the data provider possible<br>• Data already used in other publication (Publications reporting identical outcomes of the same study and no new and relevant aspects according to the research questions) → publication with more relevant information will be included<br><br>Availability:<br>• No full text available

Publication language:<br>no restriction<br><br>Availability:<br>• full text available<br>• abstracts if additional information available<br><br>Quality standards:<br>• report of n study population<br>• description of setting/study population<br>• year of data collection<br>• study tool for outcome	• Abstract with no additional information from author<br><br>Quality standards:<br>• No report of n study population<br>• No description of setting/study population<br>• No year of data collection reported<br>• No study tool for outcome reported
