# Supplementary material for: Hepatitis B vaccination coverage in Germany: systematic review
Source: BMC Infect Dis. 2021 Aug 14;21:817. doi: 10.1186/s12879-021-06400-4 (PMC8364709; doi:10.1186/s12879-021-06400-4)
Supplement: Supplementary file 2 — Additional file 2. Search strings. Search strings for search in electronical databases. [file 12879_2021_6400_MOESM2_ESM.pdf]

Search string

Search in Medline (full-text search; date of last search: 06 July 2020, search restricted to 1 January 2017 – 06 July 2020; filters: English and German language):

(("Hepatitis B"[MESH] OR hepatitis b [Title/Abstract] OR hepatitis b [Text word] OR HBV[Title/Abstract] OR HBV [Text word])
AND
(European Union [Title/Abstract]
OR EU [Title/Abstract]
OR EEA [Title/Abstract]
OR europ* [Title/Abstract]
OR germany [MESH]
OR german* [Title/Abstract]
OR Nordrhine-Westfalia [Title/Abstract]
OR Hesse [Title/Abstract]
OR Brandenburg [Title/Abstract]
OR Lower Saxony [Title/Abstract]
OR Baden-Wurttemberg [Title/Abstract]
OR Bavaria [Title/Abstract]
OR Mecklenburg-Vorpommern [Title/Abstract]
OR Rhineland-Palatinate [Title/Abstract]
OR Saarland [Title/Abstract]
OR Saxony [Title/Abstract]
OR Schleswig-Holstein [Title/Abstract]
OR Thuringia [Title/Abstract]
OR Saarland [Title/Abstract]
OR Hamburg [Title/Abstract]
OR Bremen [Title/Abstract]
OR Berlin [Title/Abstract]
OR Sachsen-Anhalt [Title/Abstract]
OR Saxony-Anhalt [Title/Abstract]
OR Mecklenburg [Title/Abstract]
OR Western Pomerania [Title/Abstract])
OR Europäische Union [Title/Abstract]
OR deutsch* [Title/Abstract]
OR Nordrhein-Westfalen [Title/Abstract]
OR Hessen [Title/Abstract]
OR Niedersachsen [Title/Abstract]
OR Baden-Württemberg [Title/Abstract]
OR Bayern [Title/Abstract]
OR Rheinland-Pfalz [Title/Abstract]
OR Sachsen [Title/Abstract]
OR Thüringen [Title/Abstract]
OR European Union [Text Word]
OR EU [Text Word]
OR EEA [Text Word]
OR europ* [Text Word]
OR german* [Text Word]
OR Nordrhine-Westfalia [Text Word]
OR Hesse [Text Word]
OR Brandenburg [Text Word]

OR Lower Saxony [Text Word]
OR Baden-Wurttemberg [Text Word]
OR Bavaria [Text Word]
OR Mecklenburg-Vorpommern [Text Word]
OR Rhineland-Palatinate [Text Word]
OR Saarland [Text Word]
OR Saxony [Text Word]
OR Schleswig-Holstein [Text Word]
OR Thuringia [Text Word]
OR Saarland [Text Word]
OR Hamburg [Text Word]
OR Bremen [Text Word]
OR Berlin [Text Word]
OR Sachsen-Anhalt [Text Word]
OR Saxony-Anhalt [Text Word]
OR Mecklenburg [Text Word]
OR Western Pomerania [Text Word]
OR Europäische Union [Text Word]
OR deutsch* [Text Word]
OR Nordrhein-Westfalen [Text Word]
OR Hessen [Text Word]
OR Niedersachsen [Text Word]
OR Baden-Württemberg [Text Word]
OR Bayern [Text Word]
OR Rheinland-Pfalz [Text Word]
OR Sachsen [Text Word]
OR Thüringen [Text Word])
AND
(prevalence [MESH] OR prevalence [Title/Abstract] OR prevalence [Text Word] OR vaccination[MESH] OR vaccin* [Title/Abstract] OR vaccin* [Text word]
OR immun* [Title/Abstract] OR immun*[tw] OR vaccination coverage [MESH] OR vaccination coverage [Title/Abstract] OR vaccination coverage [Text Word]))

Search in Embase (full-text search; date of last search: 06 July 2020, search restricted to 1 January 2017 – 06 July 2020; filters: English and German language):

(("Hepatitis B"/syn OR "Hepatitis B"/exp OR "hepatitis b":ti,ab OR "hepatitis b [Text word]" OR 'hbv':ti,ab OR 'hbv [text word]')
AND
(germany/syn
OR germany/exp
OR german*:ti,ab
OR "European Union":ti,ab
OR EU:ti,ab OR EEA:ti,ab
OR europ*:ti,ab
OR Nordrhine-Westfalia:ti,ab
OR Hesse:ti,ab
OR Brandenburg:ti,ab
OR "Lower Saxony":ti,ab
OR Baden-Wurttemberg:ti,ab
OR Bavaria:ti,ab
OR Mecklenburg-Vorpommern:ti,ab

OR Rhineland-Palatinate:ti,ab
OR Saarland:ti,ab
OR Saxony:ti,ab
OR Schleswig-Holstein:ti,ab
OR Thuringia:ti,ab
OR deutsch*:ti,ab
OR "Europäische Union":ti,ab
OR Nordrhein-Westfalen:ti,ab
OR Hessen:ti,ab
OR Niedersachsen:ti,ab
OR Baden-Württemberg:ti,ab
OR Bayern:ti,ab
OR Rheinland-Pfalz:ti,ab
OR Sachsen:ti,ab
OR Thüringen:ti,ab
OR "European Union [Text Word]"
OR "EU [Text Word]"
OR "EEA [Text Word]"
OR "europ* [Text Word]"
OR "german* [Text Word]"
OR "Nordrhine-Westfalia [Text Word]"
OR "Hesse [Text Word]"
OR "Brandenburg [Text Word]"
OR "Lower Saxony [Text Word]"
OR "Baden-Wurttemberg [Text Word]"
OR "Bavaria [Text Word]"
OR "Mecklenburg-Vorpommern [Text Word]"
OR "Rhineland-Palatinate [Text Word]"
OR "Saarland [Text Word]"
OR "Saxony [Text Word]"
OR "Schleswig-Holstein [Text Word]"
OR "Thuringia [Text Word]"
OR "deutsch* [Text Word]"
OR "Europäische Union [Text Word]"
OR "Nordrhein-Westfalen [Text Word]"
OR "Hessen [Text Word]"
OR "Niedersachsen [Text Word]"
OR "Baden-Württemberg [Text Word]"
OR "Bayern [Text Word]"
OR "Rheinland-Pfalz [Text Word]"
OR "Sachsen [Text Word]"
OR "Thüringen [Text Word]"
OR "Saarland [Text Word]"
OR "Hamburg [Text Word]"
OR "Bremen [Text Word]"
OR "Berlin [Text Word]"
OR "Sachsen-Anhalt [Text Word]"
OR "Saxony-Anhalt [Text Word]"
OR "Mecklenburg [Text Word]"
OR "Western Pomerania [Text Word]")

AND

('prevalence'/syn OR 'prevalence'/exp OR prevalence:ti,ab OR 'prevalence [text word]' OR 'vaccination'/exp OR 'vaccination'/syn OR vaccin*:ti,ab OR 'vaccination [text word]' OR 'vaccination

coverage'/exp OR 'vaccination coverage'/syn OR 'vaccination coverage':ti,ab OR 'vaccination coverage [text word]' OR immun*:ti,ab OR 'immunization [text word]' OR 'immunisation [text word]' OR prävelanz:ti,ab OR impfung:ti,ab OR impfabdeckung:ti,ab OR 'impfabdeckung [textword]' OR impfrate:ti,ab OR 'impfrate [textword]' OR impfquote:ti,ab OR 'impquote [textword]'))

Search in Livivo (full-text search; date of last search: 9 March 2017, search restricted to 1 January 2017 – 06 July 2020; filters: English and German language):

((" hepatitis b" OR HBV)
AND
(" European Union"
OR EU
OR EEA
OR europe
OR european
OR europ*
OR germany
OR german
OR german*
OR Nordrhine-Westfalia
OR Hesse
OR Brandenburg
OR Lower Saxony
OR Baden-Wurttemberg
OR Bavaria
OR Mecklenburg-Vorpommern
OR Rhineland-Palatinate
OR Saarland OR Saxony
OR Schleswig-Holstein
OR Thuringia
OR deutsch*
OR “Europäische Union”
OR Nordrhein-Westfalen
OR Hessen
OR Niedersachsen
OR Baden-Württemberg
OR Bayern
OR Rheinland-Pfalz
OR Sachsen
OR Thüringen
OR Schleswig-Holstein
OR Brandenburg
OR Mecklenburg-Vorpommern
OR Saarland
OR Hamburg
OR Bremen
OR Berlin
OR Sachsen-Anhalt
OR Saxony-anhalt
OR Mecklenburg
OR Western Pomerania)
AND

(prevalence OR vaccination OR vaccinated OR vaccin* OR immunization OR immunisation OR immunised OR immun* OR vaccination coverage OR " vaccination coverage" OR prävelanz OR impf* OR "Impfquote" OR "Impfabdeckung" OR "Impfrate"))
